# Regulation of the EGFR/ErbB signalling by clathrin in response to various ligands in hepatocellular carcinoma cell lines

**DOI:** 10.1111/jcmm.15440

**Published:** 2020-06-09

**Authors:** Yuanhui Liu, Claire Calmel, Christèle Desbois‐Mouthon, Joëlle Sobczak‐Thépot, Anthi Karaiskou, Françoise Praz

**Affiliations:** ^1^ INSERM UMR_S 938 Centre de Recherche Saint‐Antoine (CRSA) Sorbonne Université Paris France; ^2^ Centre National de la Recherche Scientifique (CNRS) Paris France

**Keywords:** clathrin, EGFR/ErbB, endocytosis, hepatocellular carcinoma, signalling, STAT3

## Abstract

Membrane receptor intracellular trafficking and signalling are frequently altered in cancers. Our aim was to investigate whether clathrin‐dependent trafficking modulates signalling of the ErbB receptor family in response to amphiregulin (AR), EGF, heparin‐binding EGF‐like growth factor (HB‐EGF) and heregulin‐1β (HRG). Experiments were performed using three hepatocellular carcinoma (HCC) cell lines, Hep3B, HepG2 and PLC/PRF/5, expressing various levels of EGFR, ErbB2 and ErbB3. Inhibition of clathrin‐mediated endocytosis (CME), by down‐regulating clathrin heavy chain expression, resulted in a cell‐ and ligand‐specific pattern of phosphorylation of the ErbB receptors and their downstream effectors. Clathrin down‐regulation significantly decreased the ratio between phosphorylated EGFR (pEGFR) and total EGFR in all cell lines when stimulated with AR, EGF, HB‐EGF or HRG, except in HRG‐stimulated Hep3B cells in which pEGFR was not detectable. The ratio between phosphorylated ErbB2 and total ErbB2 was significantly decreased in clathrin down‐regulated Hep3B cells stimulated with any of the ligands, and in HRG‐stimulated PLC/PRF/5 cells. The ratio between phosphorylated ErbB3 and total ErbB3 significantly decreased in clathrin down‐regulated cell lines upon stimulation with EGF or HB‐EGF. STAT3 phosphorylation levels significantly increased in all cell lines irrespective of stimulation, while that of AKT remained unchanged, except in AR‐stimulated Hep3B and HepG2 cells in which pAKT was significantly decreased. Finally, ERK phosphorylation was insensitive to clathrin inhibition. Altogether, our observations indicate that clathrin regulation of ErbB signalling in HCC is a complex process that likely depends on the expression of ErbB family members and on the autocrine/paracrine secretion of their ligands in the tumour environment.

## 1 INTRODUCTION

Primary liver cancers represent the sixth most frequent cancer and second leading cause of death by cancer, with 841 000 new cases and   782 000 deaths in the world in 2018.^1^ Most are hepatocellular carcinomas (HCC; 75%‐85%), mainly caused by chronic infection with hepatitis B or C viruses, excessive alcohol consumption or non‐alcoholic fatty liver disease. Numerous molecular alterations have been reported in HCC, including the epidermal growth factor receptor (EGFR) signalling pathway.^2^

EGFR, also known as ErbB1 or HER1, is the prototype member of the family of ErbB receptor tyrosine kinases that also comprises ErbB2, ErbB3 and ErbB4 (also referred as HER2, HER3 and HER4, respectively).^3^, ^4^, ^5^ ErbB receptors play key roles in the regulation of essential cellular functions notably cell proliferation and survival, differentiation and migration, by triggering multiple downstream signalling pathways, such as PI3K/AKT, MAPK/ERK, PLCγ and JAK/STAT pathways.^6^ Upon ligand binding, ErbB receptors form homo‐ or heterodimers with other family members and become transphosphorylated.^7^ ErbB2, which has no known ligand, is constitutively able to dimerize with other ligand‐bound ErbB monomers.^8^, ^9^, ^10^

The regulation of ErbB signalling depends on numerous parameters, at the membrane level and downstream. Receptor‐mediated signalling depends on the ErbB partners forming the active dimer receptor complexes, which relies on the expression levels of the various ErbB family members and on the ligands triggering receptor dimerization.^11^ EGFR has seven known ligands; four of them bind EGFR exclusively, namely, EGF, transforming growth factor α (TGFα), amphiregulin (AR) and epigen, while the three others, heparin‐binding EGF‐like growth factor (HB‐EGF), betacellulin and epiregulin are also able to bind ErbB4. EGF, TGFα, betacellulin and HB‐EGF exhibit 10‐ to 100‐fold higher affinities for EGFR than AR, epiregulin and epigen. The heregulin (HRG) family comprises four major types of HRG (HRG‐1 to 4) that bind ErbB3 and/or ErbB4, but not EGFR.^4^, ^5^

Several human malignancies have been associated with alterations of ErbB family members, most often EGFR and ErbB2.^4^, ^12^ In addition, abnormally high levels of ErbB ligands have been reported in several cancers and are suspected to promote tumour aggressiveness.^13^, ^14^, ^15^ As an example, AR is overexpressed in a wide variety of cancers, including HCC, and its role in tumour development and prognosis has been clearly established.^15^, ^16^, ^17^

ErbB cellular signalling is further regulated through endocytosis and intracellular trafficking of ligand‐bound receptors.^12^, ^18^, ^19^, ^20^, ^21^ According to the classical scheme, ligand binding to EGFR induces its internalization, followed by relocation to early endosomes and later to lysosome for degradation, or to the recycling endosome and back to plasma membrane.^22^ Clathrin‐mediated endocytosis (CME) is the main route involved in EGFR internalization.^23^, ^24^, ^25^ Clathrin is the major component of clathrin‐coated pits (CCP) that are internalized upon ligand binding, resulting in the formation of intracellular clathrin‐coated vesicles (CCV), that later fuse with early endosomes where receptors interact with several downstream effectors and continue signalling.^26^ EGFR endocytosis may also involve clathrin‐independent endocytosis (CIE) routes, depending on the ligand bound to EGFR and its concentration.^18^ The route of endocytosis plays a critical role in EGFR signalling because while CME favours EGFR recycling, CIE targets EGFR to the lysosomes where it is degraded, leading to signal extinction.^20^

The ligand itself also influences the endocytosis route involved in EGFR endocytosis and the balance between recycling and degradation. AR is mainly internalised through CME,^27^ followed by fast as well as slow recycling of EGFR to the plasma membrane, without targeting EGFR for lysosomal degradation,^28^ whereas HB‐EGF, the most potent inducer of EGFR phosphorylation efficiently stimulates CIE and targets EGFR for lysosomal degradation.^19^ However, conflicting results have been reported regarding clathrin dependency of EGFR endocytosis and intracellular trafficking reflecting that these processes may not be universal and may be submitted to fine cell‐ and condition‐dependent regulation.^29^, ^30^ Yet, it was reported in HeLa cells that internalization of EGF, added at fairly high concentrations (1.6 nmol/L), was not significantly affected by clathrin depletion, but clathrin was essential for EGF sorting to multivesicular endosomes, a key step for subsequent receptor degradation.^29^ In another study also performed in HeLa cells and its HEp2‐derived subclone, EGF uptake was strongly inhibited in clathrin‐depleted cells even at a high concentration of EGF (10 nmol/L).^31^ By contrast, ErbB2 is resistant to internalization, protecting its partner from degradation, thus leading to sustained signalling.^32^, ^33^ ErbB3 was considered to be insensitive to endocytosis until recently, when it was reported that ErbB3 is constitutively internalized in a clathrin‐dependent manner and degraded.^34^, ^35^

Alterations of endocytosis and intracellular trafficking are emerging features of cancer development and progression.^36^ High clathrin heavy chain (CHC) protein expression has been reported to be a useful marker to distinguish early HCC from benign tumours such as regenerative nodule or focal nodular hyperplasia.^37^, ^38^, ^39^ In addition, CHC expression was significantly stronger in moderately and poorly differentiated HCC tumour cells, compared to well‐differentiated tumours.^39^

Altogether, these observations prompted us to investigate the impact of clathrin expression on cell signalling in HCC cell lines exhibiting different levels of ErbB receptors and stimulated with various ligands.

## 2 MATERIALS AND METHODS

Hep3B and HepG2 cells were obtained from the American Type Culture Collection. PLC/PRF/5 cells were provided by Dr Christine Perret (Institut Cochin, Paris, France). Hep3B, HepG2 and PLC/PRF/5 HCC cell lines were maintained in Minimum Essential Medium (Life Technologies), containing Earl's salts, 25 mmol/L HEPES, 5.5 mmol/L d‐Glucose and 2 mmol/L l‐Alanyl‐Glutamine (GlutaMAX), supplemented with 10% (v/v) foetal bovine serum, 1× MEM Non‐Essential Amino Acids, 1 mmol/L sodium pyruvate, 100 U/mL penicillin and 10 µg/mL streptomycin (all additives were from Life Technologies). The absence of mycoplasma contamination was checked every month using DAPI (4′,6‐Diamidine‐2′‐phenylindole dihydrochloride) staining and fluorescent microscopy. Cell lines were regularly authenticated using the short tandem repeat (STR) panel recommended by the International Cell Line Authentication Committee comprising eight highly polymorphic tetranucleotide short tandem repeat markers (D5S818, D7S820, D13S317, D16S539, CSF1PO, THO1, TPOX and vWA) and the amelogenin marker that discriminates the X from the Y chromosome, as previously described.^40^ The STR profiles of the cell lines used were identical to those available on ATCC and DSMZ websites (Table S1). Normal human hepatocytes were isolated from liver tissue obtained from patients undergoing partial liver resection for liver metastasis, and primary cultures were established as previously described.^41^

Inhibition of CHC expression was achieved by transiently transfecting siRNA duplexes at 20 nmol/L final concentration, using Lipofectamine RNAiMax reagent (Life Technologies), following the manufacturer's protocol. Small interfering RNA for CHC (siCHC: 5′‐AACCUGCGGUCUGGAGUCAAC‐3′) and non‐targeting control siRNA (siControl: 5′‐UGGUUUACAUGUUGUGUGA‐3′) were purchased from Eurogentec. Cells were seeded into 25‐cm^2^ flasks 24 hours before transfection in antibiotics‐free medium. After 1 day, cells were subsequently divided in order to be subconfluent 4 days later when stimulation experiments were performed. The inhibitory efficiency using siCHC was assessed by analysing total cell lysates by Western blotting, as described below.

Immunofluorescence analysis of clathrin expression was performed on cells previously fixed with 4% paraformaldehyde in PBS for 10 minutes at room temperature (RT) and washed three times with phosphate‐buffered saline (PBS). Cell membranes were then permeabilized in PBS containing 0.1% TritonX‐100 for 10 minutes at RT and subsequently washed three times with PBS. Nonspecific binding was blocked with PBS containing 1% BSA for 30 minutes at RT. Cells were incubated with a rabbit polyclonal antibody specific for CHC (Abcam, ab21679, at a 1/500 dilution) overnight at 4°C, washed with PBS‐1% BSA containing 0.05% Tween‐20 and then incubated with an Alexa 488‐labeled highly cross‐adsorbed goat anti‐rabbit IgG (heavy and light chains) antibody (Invitrogen, A‐11034, diluted 1/1000) for 1 hour at RT. The coverslips were mounted on glass slides and analysed with a fluorescence microscope.

To analyse transferrin receptor endocytosis, Hep3B cells were seeded on glass coverslips and transfected with siCHC or siControl. Four days later, cells were incubated in serum‐free DMEM during 30 minutes at 37°C for transferrin starvation. Cells were then incubated in DMEM‐1% BSA‐20 mmol/L Hepes containing 25 μg/mL A488‐labeled transferrin (Molecular Probes, Life Technologies) during 1 hour at 4°C. Cells were then washed in ice‐cold DMEM‐1% BSA‐20 mmol/L Hepes and further incubated in pre‐warmed DMEM‐1% BSA‐20 mmol/L Hepes at 37°C for 10 minutes. Cells were transferred in ice‐cold PBS and to stop endocytosis process, incubated on ice for 4 minutes with cold stripping buffer (50 mmol/L Glycine, 100 mmol/L NaCl, pH 3) to remove membrane bound transferrin. Cells were then washed with PBS, fixed in 4% PFA for 15 minutes at RT and observed by confocal microscopy.

All ErbB ligands used to stimulate cells were human recombinant peptides purchased from PeproTech: EGF (AF‐100‐15), AR (100‐55B), HB‐EGF (100‐47) and HRG‐β1 (100‐03). Ligands were aliquoted and stored at −20°C until use. Before adding ErbB ligands, cells were cultured overnight in serum‐free medium, then washed and replenished with serum‐free medium for 1 hour to eliminate autocrine‐derived EGFR ligands potentially synthesized during the night. Ligands were then added to cell cultures at a final concentration of 10 nmol/L for 5 minutes at 37°C; we previously checked that phosphorylation peaked at 5 minutes, as previously reported by others.^56^

For Western blot analyses, cells were washed with PBS at 4°C, lysed with RIPA buffer containing 0.1% SDS, 1% NP40, 150 mmol/L NaCl and 50 mmol/L Tris‐HCl pH 7.5 supplemented with sodium deoxycholate (0.5%, Merck), Halt Protease & Phosphatase Inhibitor Cocktail 2× (#78442, Pierce, Thermo Scientific) and 10 mmol/L EDTA and further kept on ice. Protein concentrations were assessed using the BCA protein assay (#23227, Pierce, Thermo Scientific), according to the protocol of the manufacturer. Fifteen micrograms of total proteins were then loaded into each well of a TGX Stain‐Free precast 4%‐15% acrylamide gradient gel (Bio‐Rad). Samples were then resolved by SDS‐PAGE and transferred onto a 0.2 µm nitrocellulose membrane (Trans‐Blot^®^ Turbo™ Transfer System, Bio‐Rad). In order to avoid problems related to signal normalization using housekeeping proteins, normalization was performed by measuring total protein in each lane on the membrane using stain‐free imaging technology (Bio‐Rad). This technique enabled us to visualize UV‐induced fluorescence of SDS‐PAGE gels and their corresponding blots using a ChemiDoc Touch Imaging system (Bio‐Rad). The relative amount of total protein in each lane on the blot was calculated with Image Lab software (Bio‐Rad) and used for quantitation normalization.^42^

Membranes were first blocked with 5% bovine serum albumin (BSA) for 1 hour, and then incubated overnight at 4°C in 5% BSA containing primary antibodies. Unless otherwise stated, primary antibodies were monoclonal rabbit antibodies purchased from Cell Signaling Technology and used at a 1/1000 dilution: CHC (ab‐21679, Abcam), pEGFR‐Y1068 (#3777), EGFR (#4267), pErbB2‐Y1221/Y1222 (#2243), ErbB2 (#2165), pErbB3‐Y1289 (#4791), ErbB3 (#4754), pSTAT3‐Y705 (#9145), STAT3 (#4904), pAKT‐S473 (#4060, 1/500), AKT (#4691), pERK‐T202/Y204 (#4370), and ERK (sc‐93, 1/250, rabbit polyclonal, Santa Cruz Biotechnology). Stain‐free total protein staining technology was used as a loading control, as it was shown to be more consistent than housekeeping proteins.^42^ After washing three times in TBST 1× containing 20 mmol/L Tris‐buffered saline—0.2% Tween 20—136 mmol/L NaCl, horse radish peroxidase‐conjugated anti‐rabbit (#7074) or anti‐mouse (#7076) secondary antibodies from Cell Signaling Technology were added at 1/5000 in PBS containing 5% BSA and incubated for 1 hour at room temperature, washed three times, before addition of Clarity Western ECL substrate (170‐5061, Bio‐Rad). In most cases, the membranes were first probed with antibodies specific for the phosphorylated proteins, stripped and then probed with antibodies against the corresponding protein. When the quality of the membranes was not good enough after stripping (in particular when signals were strong), the corresponding protein extracts were reloaded onto a new gel and probed directly with the appropriate antibody. Membrane signals were detected and quantified using the ChemiDoc Touch Imaging System and Image Lab software (Bio‐Rad). Signal intensities were normalized to the total protein quantity loaded per lane. Quantitative data represent the mean of the ratio of the normalized signal intensities measured in cells transfected with siCHC relative to cells transfected with non‐targeting siRNA, obtained in at least three independent experiments. Blots showing conditions leading to statistically different phosphorylation levels are shown in dashed boxes.

The ratios of the signal intensities obtained in cells transfected with siCHC relative to siControl were compared using a two‐sided unpaired *t* test, with Welch's correction if variances were significantly different. Histograms representing relative phosphorylation levels are shown when at least one statistically significant variation of the signals was observed for a given cell line; *P* values are indicated by * for *P* < .05, ** for *P* < .01 and *** for *P* < .001.

## 3 RESULTS

**FIGURE 1:**
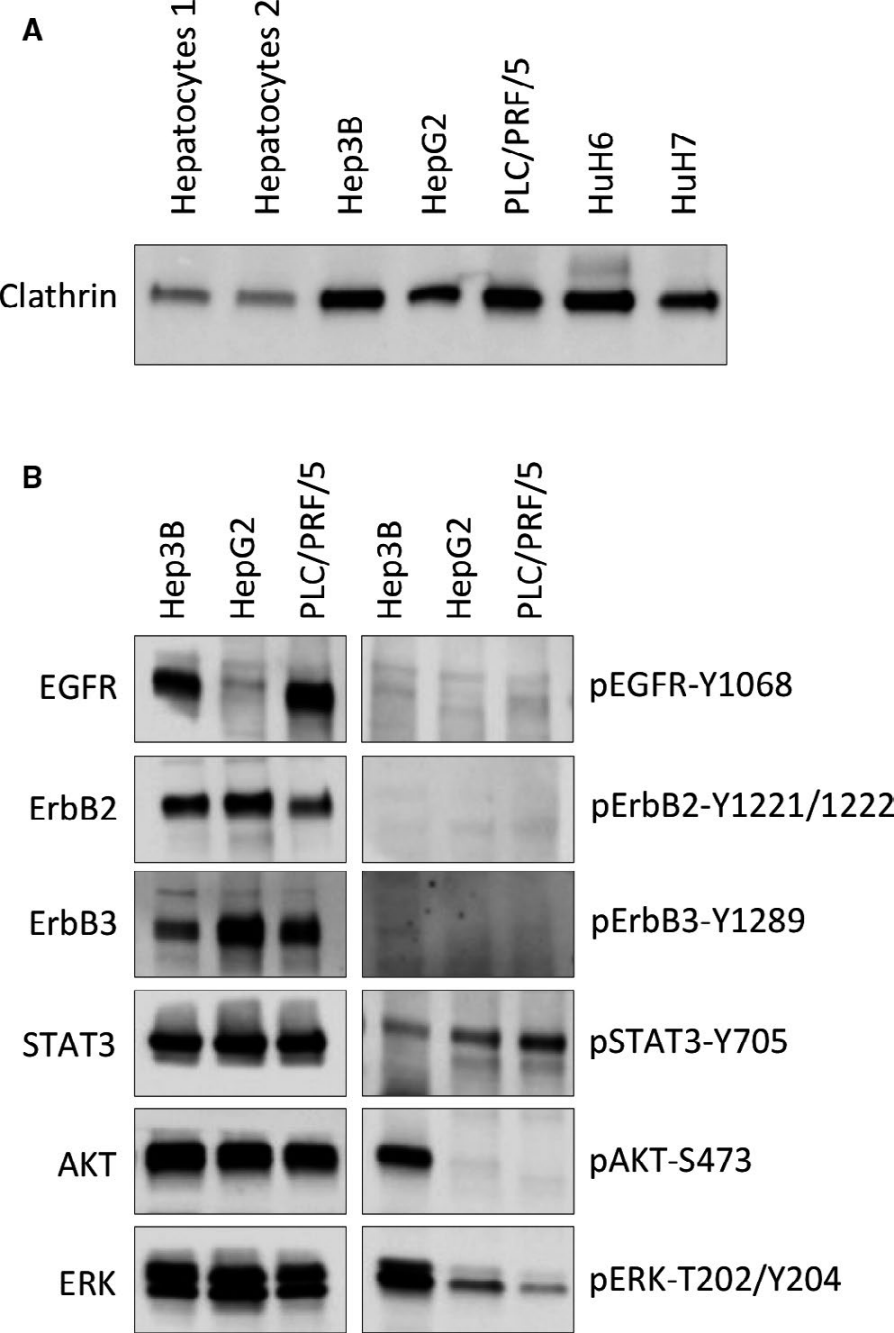
Expression of clathrin, ErbB family members and downstream signalling targets in various HCC cell lines, and their basal phosphorylation levels in the absence of ligand stimulation. Clathrin expression levels were analysed by Western blotting using total protein lysates (20 µg) obtained from Hep3B, HepG2, PLC/PRF/5, HuH6 and HuH7 cell lines, and two independent preparations of normal hepatocytes (A). ErbB family members and downstream signalling targets were analysed in HCC cell lines previously cultured overnight in absence of serum (B). Membranes were probed with antibodies specific for clathrin (A), EGFR, pEGFR‐Y1068, ErbB2, pErbB2‐Y1221/1222, ErbB3, pErbB3‐Y1289, STAT3, pSTAT3‐Y705, AKT, pAKT‐S473, ERK and pERK‐Thr202/Tyr204 (B), as indicated

The expression levels of CHC, analysed by Western blot, were much higher in HCC cell lines compared to normal hepatocytes and varied between cell lines (Figure 1A). In order to define the role of clathrin in EGFR signalling, we selected three well‐characterized cell lines expressing clathrin at comparable levels, namely Hep3B, HepG2 and PLC/PRF/5. Whole‐exome RNA and microRNA sequencing, as well as quantification of 126 proteins in 34 HCC cell lines, including those used in this study are available at http://lccl.zucmanlab.com/hcc/home. Briefly, Hep3B, derived from an HCC, carries 409 mutations and copy number alterations (CNAs), including a homozygous *TP53* deletion and integrated hepatitis B virus (HBV). HepG2, derived from an hepatoblastoma, displays 299 mutations and CNAs including *CTNNB1* exon 3 deletion and NRAS missense mutation. PLC/PRF/5, derived from an HCC, with 901 mutations and CNAs including *TP53*, *AXIN1* and *ARID1A* mutations, and secreting HBsAg. The expression levels of ErbB receptors varied among these three HCC cell lines (Figure 1B, left panels). EGFR was expressed at the highest level in PLC/PRF/5 cells, while being hardly detectable in HepG2 cells. ErbB2 was expressed at a slightly higher level in HepG2 cells compared to Hep3B and PLC/PRF/5 cells, which expressed the lowest level. ErbB3 was also expressed in all three cell lines analysed, with the highest and lowest levels observed in HepG2 and Hep3B cells, respectively. There was no detectable basal phosphorylation of EGFR‐Y1068, ErbB2‐Y1221/Y1222 and ErbB3‐Y1289 in these cell lines cultured in absence of serum (Figure 1B, right panels).

The well‐known downstream targets of the ErbB signalling pathways, STAT3, AKT and ERK, were expressed at comparable levels in all three cell lines (Figure 1B, left panels). As already reported by us and others, pAKT‐S473 and pERK‐T202/Y204 phosphorylation was observed in Hep3B cells, in the absence of exogenously added ErbB ligands.^43^, ^44^, ^45^ Basal pSTAT3‐Y705 phosphorylation was also detected in all three cell lines, with PLC/PRF/5 displaying the highest signal (Figure 1B, right panels).

**FIGURE 2:**
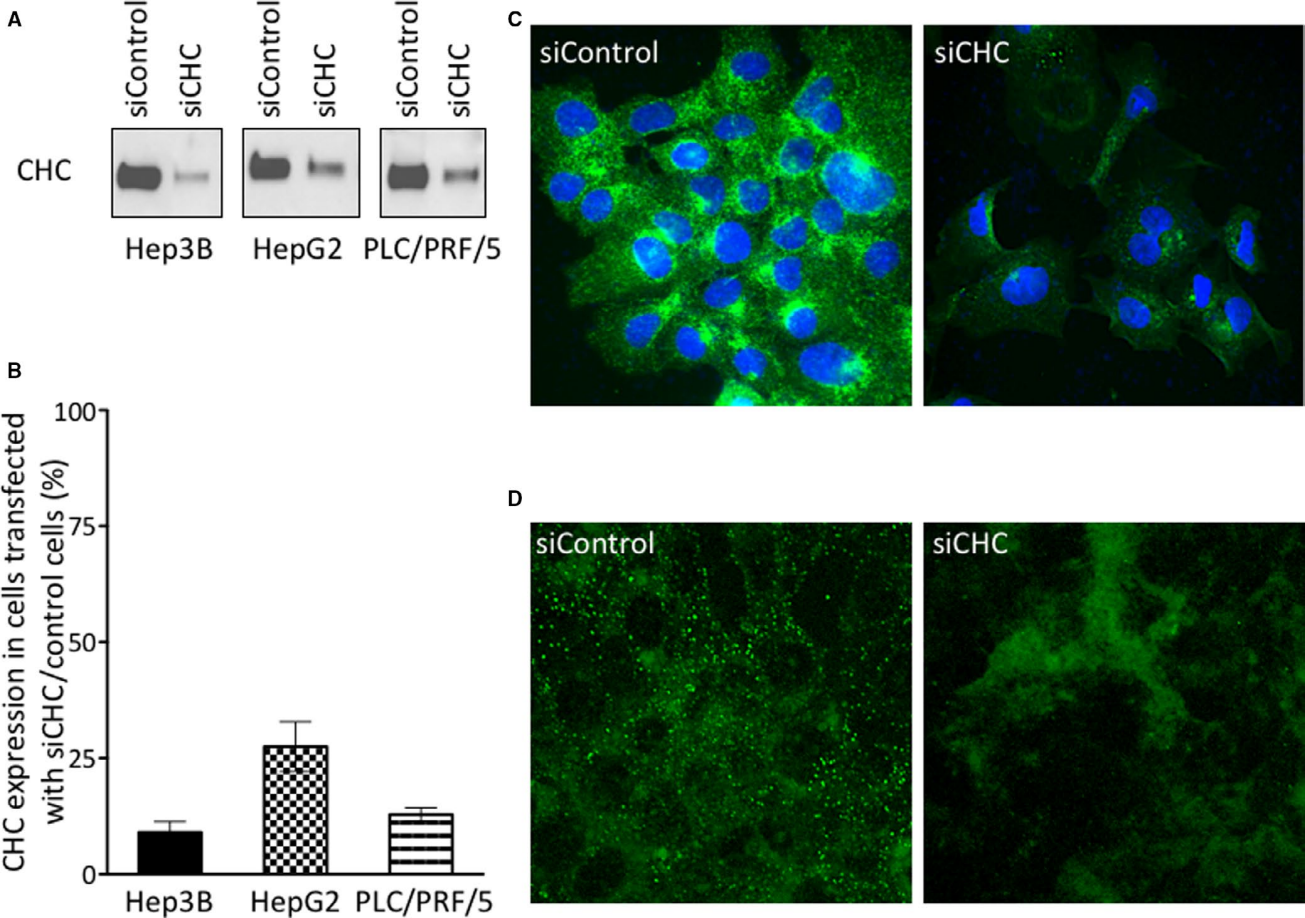
Inhibition of clathrin expression by siRNA in HCC cell lines. Hep3B, HepG2 and PLC/PRF/5 cells were transfected with non‐targeting siRNA (siControl) or siRNA specific for clathrin heavy chain (siCHC), and harvested after 120 h, as described. Total cell lysates (20 µg/lane) were analysed by Western blotting with an anti‐CHC antibody (A). Densitometric analyses of the blots obtained for at least three independent experiments per cell line have been performed using ChemiDoc Touch Imaging system. Stain‐free signals were measured on the whole lanes using Image Lab software (Bio‐Rad) and were used for quantitation normalization in order to calculate the relative signal intensity in each condition. The ratios of CHC expression in cells transfected with siCHC relative to siControl cells were calculated; histograms represent means ± SEM (B). Hep3B cells transfected with siControl (C, left panel) or siCHC (C, right panel) were fixed, permeabilized and stained with an antibody for clathrin heavy chain (green). Nuclei were stained with DAPI (blue). Endocytosis of transferrin receptors was analysed in Hep3B cells transfected with siControl (D, left panel) or siCHC (D, right panel) after a 10‐min incubation at 37°C with Alexa‐488 labelled transferrin, as described in Materials and Methods

Clathrin modulation of ErbB signalling in the selected HCC cell lines was assessed by transfection with siRNA to CHC or with non‐targeting control siRNA. CHC levels were gauged by Western blotting of whole cell lysates harvested from day 2 to day 6 after transfection. Clathrin expression levels started to decrease significantly at day 3 and maximal inhibition was reached between day 4 and day 6 (data not shown). The cell viability after siCHC was not affected during the first 3 days and started to decrease by a maximum of 30% at day 4. The best clathrin inhibition efficiency was in Hep3B (90 ± 4%) and PLC/PRF/5 cells (87 ± 3%), as compared to HepG2 (72 ± 11%; Figure 2A,B). Immunofluorescence analysis of CHC showed that Hep3B cells displayed a punctate pattern of clathrin staining, with fluorescence being preferentially condensed in the juxtanuclear region of the cells (Figure 2C; left panel). Five days after transfection with siCHC, the overall clathrin fluorescence magnitude in Hep3B cells, was much lower as compared to cells transfected with non‐targeting siControl (Figure 2C, right panel). The effect of CHC depletion on CME, was evaluated using fluorescent transferrin, which is a prototype molecule internalized by CME. Transferrin remains bound to its receptor during the whole endocytosis process, that is internalization and recycling, thus allowing the location and distribution of the receptor‐ligand complex to be followed. As shown in Figure 2D, after a 10‐min incubation with fluorescent transferrin, control cells showed a typical punctate distribution of transferrin receptors. By contrast, CHC‐depleted cells displayed a uniform widespread fluorescent signal, indicating that transferrin‐bound receptors did not relocate in CCV, thus impeding CME.

**FIGURE 3:**
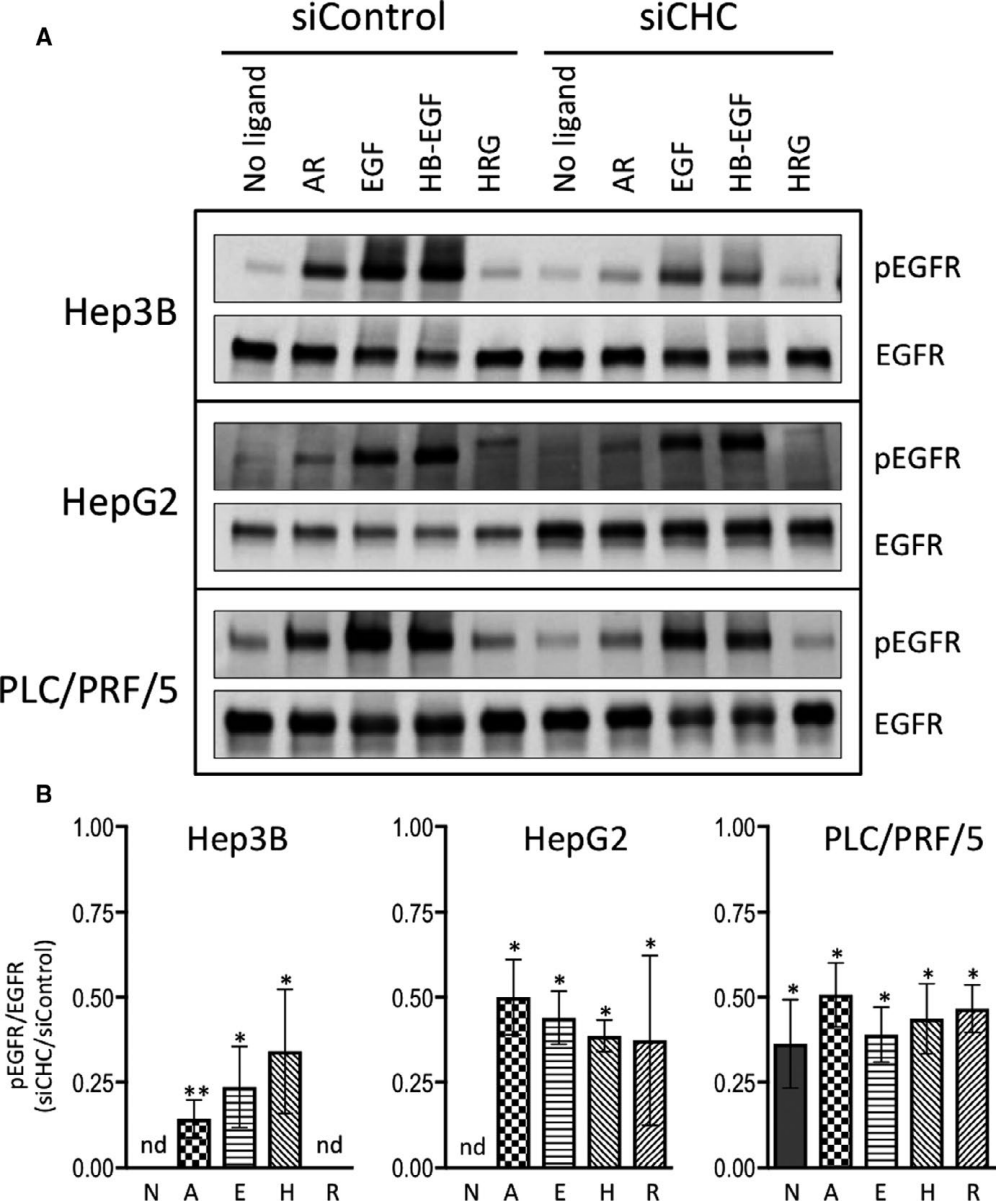
Impact of clathrin inhibition on phosphorylation of EGFR at Y1068 upon stimulation with various ligands. Hep3B, HepG2 and PLC/PRF/5 cells transfected with non‐targeting siRNA (siControl) or siRNA specific for CHC (siCHC) for 5 d. Cells were cultured in serum‐free medium for the last 16 h and subsequently treated or not with 10 nmol/L AR, EGF, HB‐EGF or HRG for 5 min at 37°C. Total protein lysates were analysed by Western blotting with antibodies against pEGFR‐Y1068 and total EGFR (A). The blot is representative of at least three independent experiments. The stain‐free signals were used as loading controls to normalize the data (not shown). The results of the densitometric analyses of pEGFR‐Y1068 relative to total EGFR in Hep3B cells, HepG2 and in PLC/PRF/5 cells obtained in three independent experiments are shown in panel B; the legend is as follow: N for no ligand, A for AR, E for EGF, H for HB‐EGF, R for HRG. Histograms represent the means ± SEM of the ratio between the signals obtained in cells transfected with siCHC relative to cells transfected with siControl. Statistical analyses were performed using the two‐sided *t* test. "*" and "**" indicate *P* values below 0.05 and 0.01, respectively; "nd" for "not determined" means that the signal intensities were too low to be adequately quantified

Four days after transfection with a CHC‐specific siRNA or a non‐targeting control siRNA, cells were serum starved overnight, followed by a 1 hour incubation in fresh serum‐free culture medium, and subsequently stimulated for 5 minutes at 37°C with ligands at a 10 nmol/L final concentration. Expression levels of EGFR and pEGFR‐Y1068 were analysed by Western blotting on whole cell lysates. In all three cell lines, EGF and HB‐EGF were the most powerful inducers of EGFR phosphorylation, whether clathrin expression was reduced or not, while phosphorylation induced by HRG was hardly detectable (Figure 3A).

Reduced clathrin expression was associated with a significant twofold to fivefold decrease in the ratio between phosphorylated EGFR and total EGFR levels, in all three cell lines and in response to all ligands (Figure 3B); such a decrease was also observed in unstimulated PLC/PRF/5 cells in which EGFR phosphorylation may be detected in absence of exogenously added ligand. Transfection of siCHC in HepG2 was associated with a twofold to threefold highly significant increase in EGFR expression, whatever cells were stimulated with any of the ErbB ligands or not, indicating that although inhibition of CHC expression was only partial, it was sufficient to modulate EGFR expression (Figure 3A and Figure S1).

**FIGURE 4:**
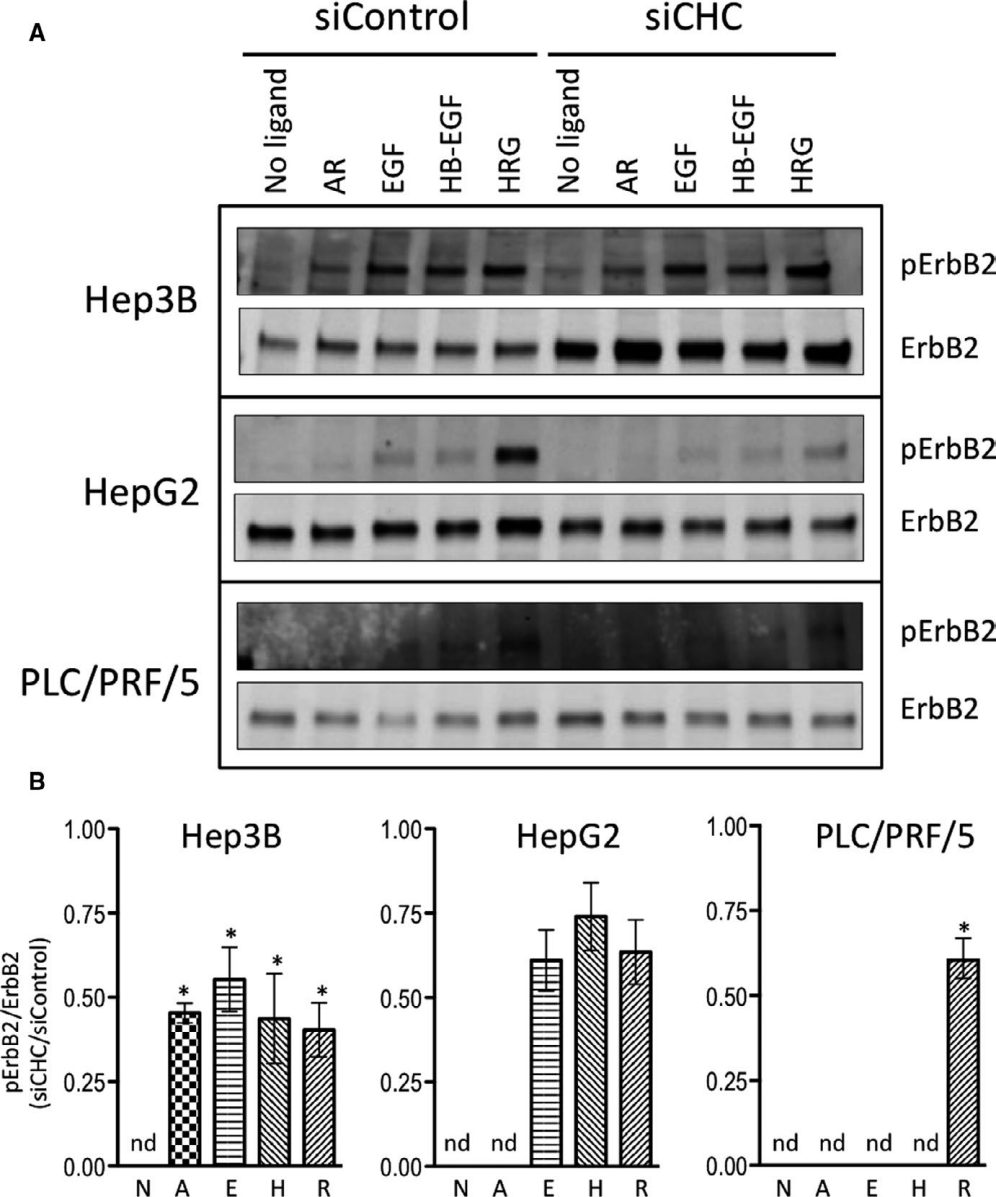
Impact of clathrin inhibition on phosphorylation of ErbB2 at Y1221/1222 upon stimulation with various ligands. Hep3B, HepG2 and PLC/PRF/5 cells transfected with non‐targeting siRNA (siControl) or siRNA specific for CHC (siCHC) for 5 days. Cells were cultured in serum‐free medium for the last 16 h and subsequently treated or not with 10 nM AR, EGF, HB‐EGF or HRG for 5 min at 37°C. Total protein lysates were analysed by Western blotting with antibodies against pErbB2‐Y1221/1222 and total ErbB2 (A). The blot is representative of at least three independent experiments. The stain‐free signals were used as loading controls to normalize the data (not shown). The results of the densitometric analyses of pErbB2‐Y1221/1222 relative to total ErbB2 in Hep3B cells, HepG2 and in PLC/PRF/5 cells obtained in three independent experiments are shown as histograms representing the means ± SEM of the normalized signal intensities (B); the legend is as follow: N for no ligand, A for AR, E for EGF, H for HB‐EGF, R for HRG. Statistical analyses were performed using the two‐sided *t* test. "*" indicates a *P* value below 0.05; "nd" for "not determined."

Regarding ErbB2 activation, HRG was the most efficient inducer of ErbB2 phosphorylation at Y1221/1222 in all three cell lines (Figure 4A). In Hep3B cells, ErbB2 phosphorylation was also efficiently induced upon stimulation with EGF or HB‐EGF, though to a fairly lower extent. The ratio between phosphorylated ErbB2 and total ErbB2 was significantly affected by clathrin inhibition in Hep3B and in PLC/PRF/5 cells, being reduced to about half under all measurable conditions (Figure 4B). Clathrin inhibition also decreased ErbB2 phosphorylation in HepG2 cells, but the effects were less marked and failed to reach statistical significance. In addition, total ErbB2 expression was increased by 2.5‐fold to fourfold in clathrin‐depleted Hep3B cells (Figure 4A and Figure S1), but its expression did not vary upon stimulation.

**FIGURE 5:**
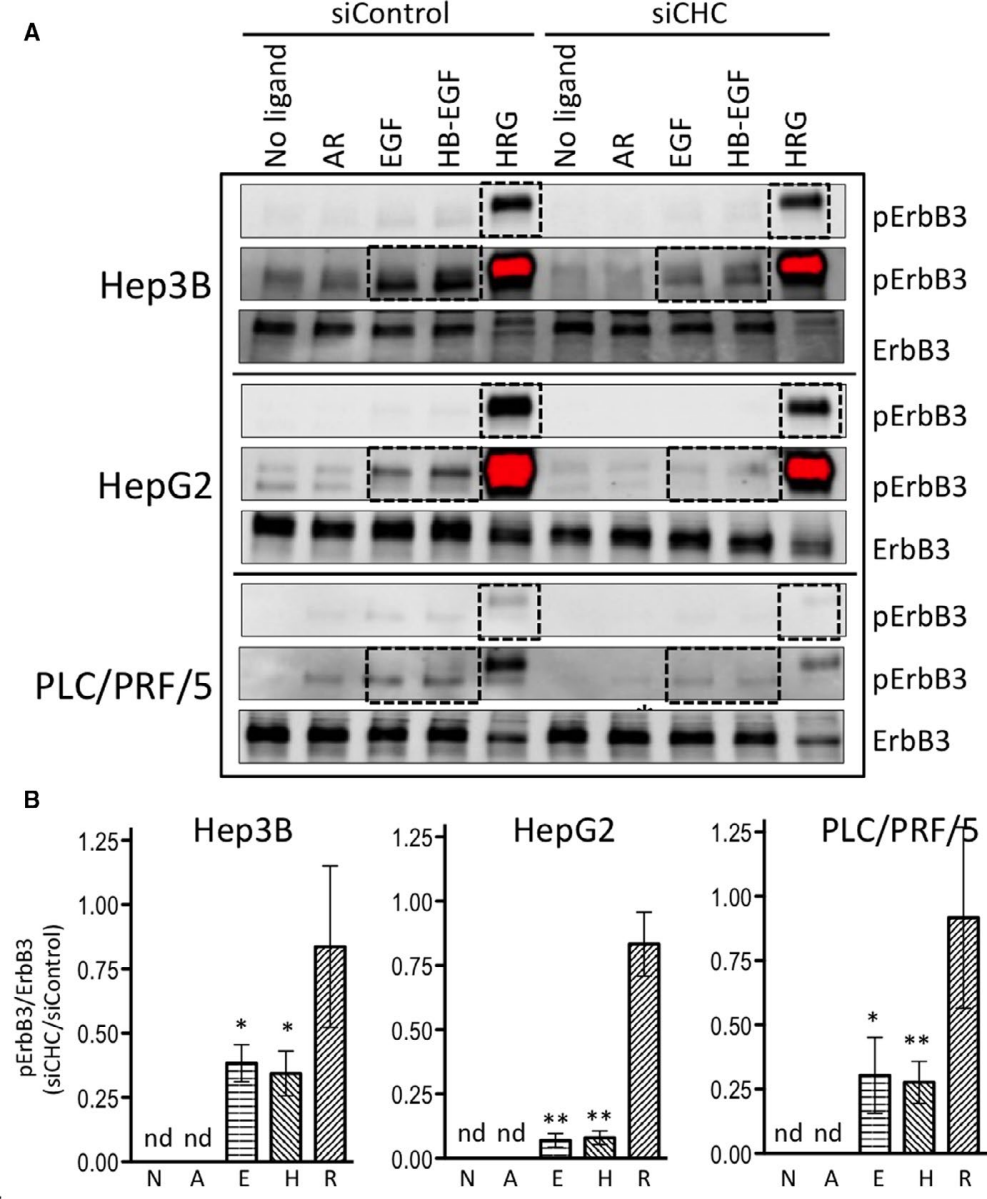
Impact of clathrin inhibition on phosphorylation of ErbB3 at Y1289 upon stimulation with various ligands. Hep3B, HepG2 and PLC/PRF/5 cells transfected with non‐targeting siRNA (siControl) or siRNA specific for CHC (siCHC) for 5 d. Cells were cultured in serum‐free medium for the last 16 h and subsequently treated or not with 10 nmol/L AR, EGF, HB‐EGF or HRG for 5 min at 37°C. Total protein lysates were analysed by Western blotting with antibodies against pErbB3‐Y1289 and total ErbB3 (A). The blot is representative of at least three independent experiments. The stain‐free signals were used as loading controls to normalize the data (not shown). The results of the densitometric analyses of pErbB3‐Y1289 relative to total ErbB3 in Hep3B cells, HepG2 and in PLC/PRF/5 cells obtained in three independent experiments are shown as histograms representing the means ± SEM of the normalized signal intensities (B); the legend is as follow: N for no ligand, A for AR, E for EGF, H for HB‐EGF, R for HRG. Because HRG‐induced ErbB3 phosphorylation is much stronger that that induced by EGF and HB‐GF, signals were quantified on the same membrane at different exposure times; spots that were measured are in dashed line rectangle. Statistical analyses were performed using the two‐sided *t* test. "*" and "**" indicate *P* values below 0.05 and 0.01, respectively; "nd" for "not determined."

ErbB3 phosphorylation was efficiently induced by HRG in the three cell lines (Figure 5A). None of the other ligands tested were able to significantly induce ErbB3 phosphorylation, though phosphorylation signals in response to EGF or HB‐EGF could be detected on longer membrane exposures (Figure 5A). Upon stimulation with EGF or HB‐EGF, down‐regulation of clathrin significantly decreased ErbB3 phosphorylation levels in all three cell lines (Figure 5B), whereas HRG‐induced ErbB3 phosphorylation was not significantly affected by clathrin expression down‐regulation (Figure 5B).

**FIGURE 6:**
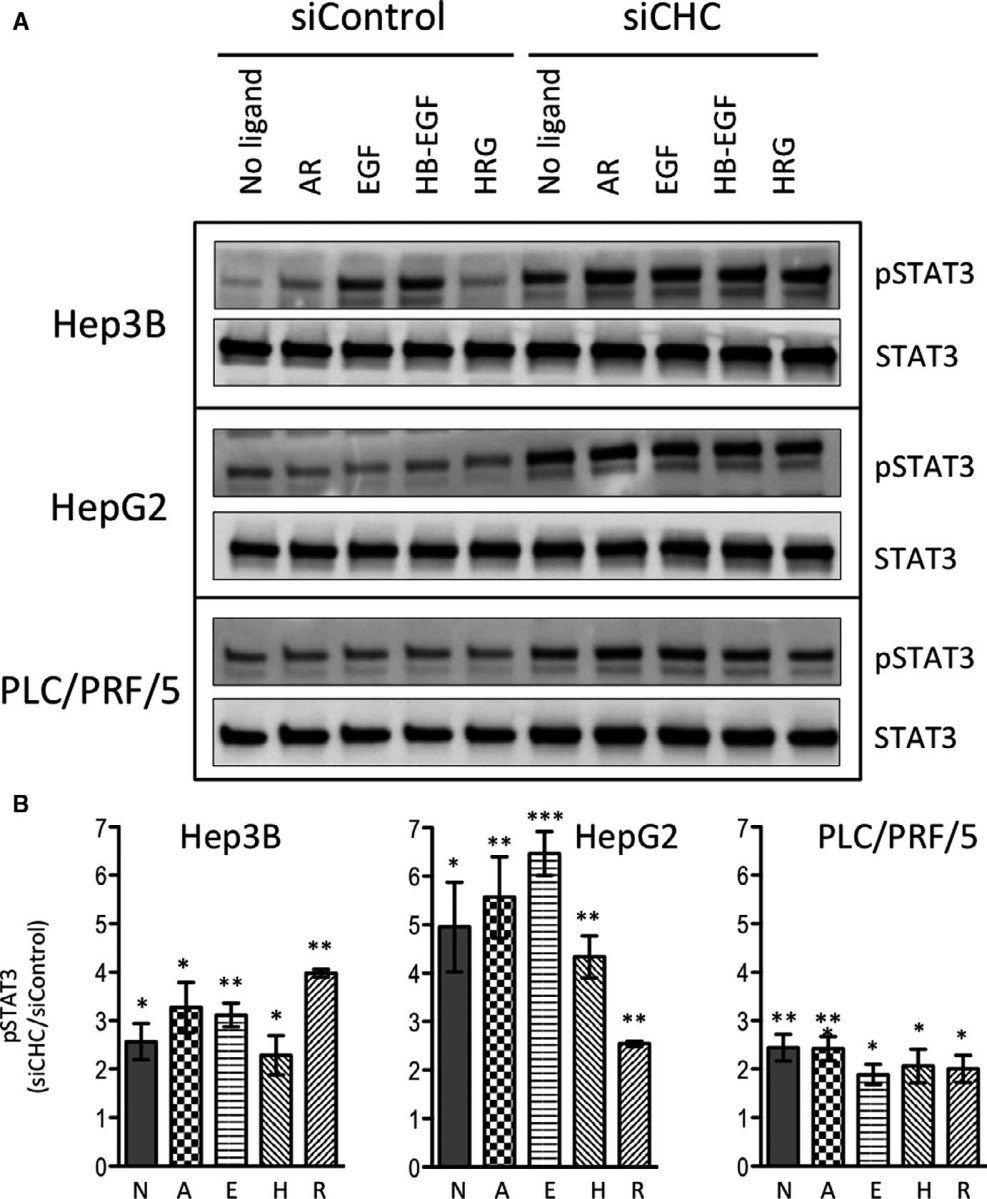
Impact of clathrin inhibition on STAT3 phosphorylation upon stimulation with various ligands. Cells were treated and analysed as in Figure 3, except that membranes were probed with antibodies specific for pSTAT3‐Y705 and total STAT3 (A). The results of the densitometric analyses of pSTAT3‐Y705 in Hep3B, in HepG2 and in PLC/PRF/5 cells are shown as histograms representing the means ± SEM of the normalized signal intensities obtained in three independent experiments (B). Statistical analyses were performed using the two‐sided *t* test. "*", "**" and "***" indicate *P* values below 0.05, 0.01 and 0.001, respectively

STAT3 was constitutively phosphorylated in HepG2 and PLC/PRF/5 cells, at levels that could not be further increased by the addition of any of the ErbB ligands tested (Figure 6A). In Hep3B cells, STAT3 phosphorylation increased over the basal level by adding EGF or HB‐EGF (Figure 6A). In clathrin‐depleted cells, STAT3 was constitutively phosphorylated, and phosphorylation levels remained unchanged upon addition of the ligands (Figure 6A). Upon inhibition of clathrin expression, pSTAT3‐Y705 signals significantly increased by twofold to sixfold in all cell lines, whatever cells were stimulated or not (Figure 6B).

**FIGURE 7:**
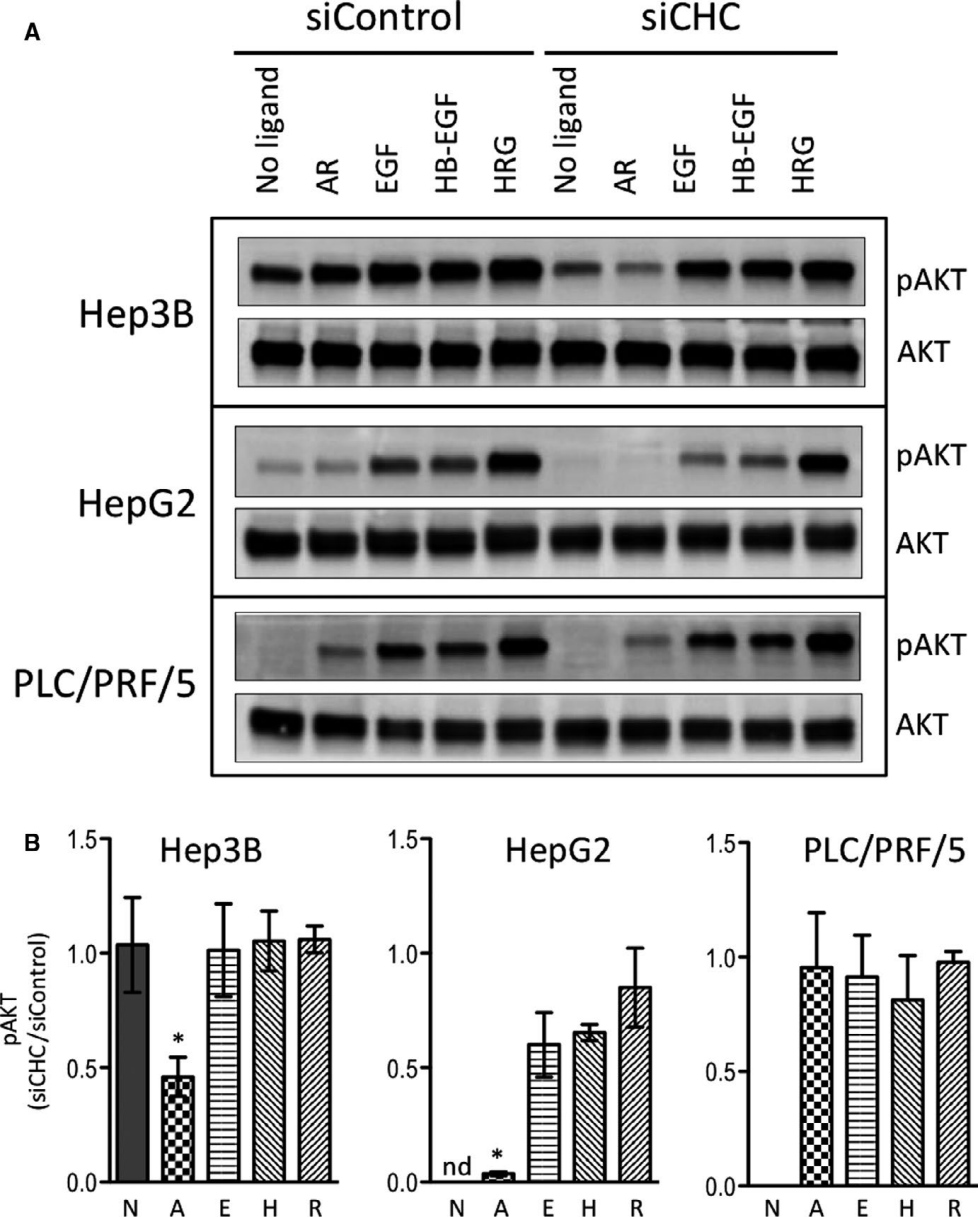
Impact of clathrin inhibition on AKT phosphorylation upon stimulation with various ligands. Cells were treated and analysed as in Figure 3, except that membranes were probed with antibodies specific for pAKT‐S473 and total AKT (A). The results of the densitometric analyses of pAKT‐S473 in Hep3B, in HepG2 and in PLC/PRF/5 cells are shown as histograms representing the means ± SEM of the normalized signal intensities obtained in three independent experiments (B). Statistical analyses were performed using the two‐sided *t* test. “*” indicates *P* values below 0.05; “nd,” not determined; “ns,” not significant

In all three cell lines, pAKT‐S473 phosphorylation levels increased upon addition of ligands, with HRG being the strongest inducer and AR the weakest one (Figure 7A). As previously reported,^45^ AKT was phosphorylated in Hep3B, in absence of exogenously added ErbB ligands. The only significant effects of clathrin inhibition were observed in Hep3B and HepG2 cells stimulated with AR in which it induced a significant decrease in AKT phosphorylation (Figure 7B). AKT phosphorylation was insensitive to inhibition of clathrin expression in all other conditions analysed (Figure 7B).

Regarding ERK activation, all three cell lines behaved similarly. ERK phosphorylation was equally induced by all ligands tested, except for AR that was less efficient in HepG2 and in PLC/PRF/5 cells, compared to the other ligands tested. In addition, ERK phosphorylation was insensitive to down‐regulation of clathrin expression (Figure S2).

## 4 DISCUSSION

Based on the previous reports showing the links between EGFR trafficking and signalling, we investigated the effects of clathrin inhibition on ErbB signalling in three HCC cell lines stimulated with various ligands. Among the EGFR ligands described so far, we analysed the effects of three of them, AR, EGF and HB‐EGF, as they were shown to regulate EGFR endocytosis and trafficking differently.^19^, ^27^ It has been reported that CME favours recycling of the receptors, whereas CIE drives them to the lysosome where they are degraded.^18^ The formation of the homo‐ or heterodimeric ErbB receptor combinations depends not only on the ligand, but also on the expression levels of the various ErbB family members. Thus, in order to investigate clathrin dependency of the various receptor dimers, we used three HCC cell lines, Hep3B, HepG2 and PLC/PRF/5, that express varying levels of EGFR, ErbB2 and ErbB3, with no detectable ErbB4 expression, thus being representative of most HCC cell lines.

EGF and HB‐EGF, the most potent inducers of receptor phosphorylation in our study, bind to both EGFR homodimers and EGFR/ErbB2 heterodimers,^4^, ^46^ with ErbB2 being the preferred dimerization partner of EGFR.^32^, ^46^, ^47^ EGFR homodimers are endocytosed rapidly after EGF stimulation, which is an indispensable step for receptor degradation, whereas ErbB2 is resistant to endocytosis, remaining at the plasma membrane together with its partner EGFR, thus hampering its degradation.^10^, ^33^, ^48^, ^49^, ^50^ The fact that the three HCC cell lines express significant levels of ErbB2 may explain why no significant EGFR degradation was observed. In addition, a 5‐min stimulation is too short to induce significant degradation, which is classically observed after 1 hour.^51^, ^52^ EGFR phosphorylation induced by AR was much weaker than with EGF or HB‐EGF, in all cell lines analysed, which is not surprising as AR has a much lower affinity for EGFR than the other ligands and displays similarly low affinity for EGFR homodimers and EGFR/ErbB2 heterodimers.^27^, ^46^ Our data showing that HRG efficiently induced phosphorylation of ErbB2 and ErbB3 in all cell lines are in accordance with the fact that both these receptors are expressed at significant levels in the HCC cell lines studied, and with the previous reports showing that HRG binds ErbB3 whose preferred partner is ErbB2.^53^, ^54^ In the presence of EGFR, ErbB2 and ErbB3, ErbB2/ErbB3 dimers are more efficiently formed than EGFR/ErbB3 dimers in response to HRG.^54^ It was recently reported that EGFR/ErbB3 can be activated upon stimulation with EGF or HRG, but the pattern of receptor phosphorylation differed significantly depending on the ligand: EGFR was phosphorylated at Y1068 when cells were stimulated with EGF, but not in the presence of HRG, as observed in our study.^55^ However, in our experiments, only HRG efficiently induced ErbB3 phosphorylation at Y1289, which contrasts with a previous report where both EGF‐ and HRG‐efficient inducers.^55^ As ligand concentrations and kinetics were the same, such discrepancy may be attributable to the use of different cellular models, namely an EGFR‐null clone of murine NIH/3T3 fibroblasts stably transfected with plasmids expressing chimeric receptors, compared to unmodified human HCC cell lines.

In our experiments, the major effects observed in response to down‐regulation of clathrin expression was a significant decrease in the phosphorylation levels of all receptors, EGFR, ErbB2 and ErbB3, associated with a significant increase in STAT3 phosphorylation, while AKT phosphorylation decreased in cells stimulated with AR, but not with the other ligands tested. Thus, our results are in apparent contradiction with the recently published data investigating the effects of clathrin inhibition on EGFR and AKT signalling using retinal pigment epithelial ARPE‐19 cells, which express low levels of ErbB2.^56^ In these cells, clathrin inhibition by siRNA did not modify EGF‐induced phosphorylation of EGFR on Y1068, but ablated AKT phosphorylation.^56^ Interestingly, EGF‐induced AKT phosphorylation became insensitive to clathrin inhibition when ErbB2 was stably expressed in these cells.^56^ Yet, a decrease in EGF‐induced AKT phosphorylation had previously been reported in clathrin‐depleted HeLa cells that are known to express ErbB2,^18^ but this study focused on the effects of clathrin on sustained EGFR signalling, later than 30 minutes after EGF addition,^18^ whereas Garay and colleagues^56^ investigated early responses occurring between 5 and 10 minutes after ligand addition, as in our study. Altogether, these observations led us to conclude that our inability to observe a significant impact of clathrin inhibition on AKT phosphorylation upon addition of EGF reflects the fact that all HCC cell lines studied expressed ErbB2.

Finally, our study shows that clathrin depletion enhanced STAT3 phosphorylation in all three HCC cell lines analysed, whatever they were stimulated with ErbB ligands or not. Contradictory effects of clathrin depletion had been reported, which either favoured NF‐κB nuclear location and activation,^57^ or attenuated its signalling.^58^ One may postulate that the increase in STAT3 phosphorylation observed in our experiments results from the inhibition of NF‐κB activation induced by clathrin depletion, thus hampering NF‐kB to play its negative control on STAT3 activation. Phosphorylation of STAT3 on Y705 has been reported to promote its nuclear translocation and subsequent transcriptional activity.^59^ It was further reported that STAT3 translocation from the cytoplasm to the nucleus requires receptor‐mediated endocytosis, but the involvement of clathrin in this process has not been investigated.^60^ Whether the increased levels of ErbB2 and EGFR, observed respectively in clathrin‐depleted Hep3B and HepG2 cells, results from an increased transcriptional activity of pSTAT3 remains to be investigated.

In conclusion, clathrin down‐regulation decreases phosphorylation of ErbB receptors in HCC cell lines, in a way that depends on the ligand used to stimulate cells and on their pattern of ErbB receptor expression. Clathrin down‐regulation further significantly decreased AKT phosphorylation in response to AR, a ligand that is frequently produced at very high levels in the tumour environment of HCC, but increased STAT3 phosphorylation irrespective of whether cells were stimulated or not. More experiments are needed to get better insights into the relationships between ErbB signalling and intracellular trafficking in HCC, in order to improve, ultimately, the therapeutic strategies based on targeting the ErbB receptor family.

## CONFLICT OF INTEREST

The authors confirm that there are no conflicts of interest.

## AUTHOR CONTRIBUTIONS

**Yuanhui Liu:** Data curation (lead); Formal analysis (lead); Investigation (lead); Methodology (equal); Validation (equal); Writing‐review & editing (equal). **Claire Calmel:** Data curation (equal); Investigation (equal); Methodology (equal); Validation (equal). **Christèle Desbois‐Mouthon:** Conceptualization (supporting); Formal analysis (supporting); Methodology (supporting); Validation (supporting); Writing‐review & editing (supporting). **Joëlle Sobczak‐Thépot:** Data curation (supporting); Investigation (supporting); Methodology (supporting); Validation (supporting). **Anthi Karaiskou:** Data curation (supporting); Investigation (supporting); Methodology (supporting); Validation (supporting). **Françoise PRAZ:** Conceptualization (lead); Data curation (lead); Formal analysis (lead); Funding acquisition (lead); Investigation (lead); Methodology (lead); Project administration (lead); Resources (lead); Supervision (lead); Validation (lead); Visualization (lead); Writing‐original draft (lead); Writing‐review & editing (equal).

## Supporting information

Fig S1

Fig S2

Table S1

## Data Availability

The data that support the findings of this study are available from the corresponding author upon reasonable request.
