# Supplementary figures and images for: Regulation of the EGFR/ErbB signalling by clathrin in response to various ligands in hepatocellular carcinoma cell lines
Source: J Cell Mol Med. 2020 Jun 9;24(14):8091–102. doi: 10.1111/jcmm.15440 (PMC7348188; doi:10.1111/jcmm.15440)

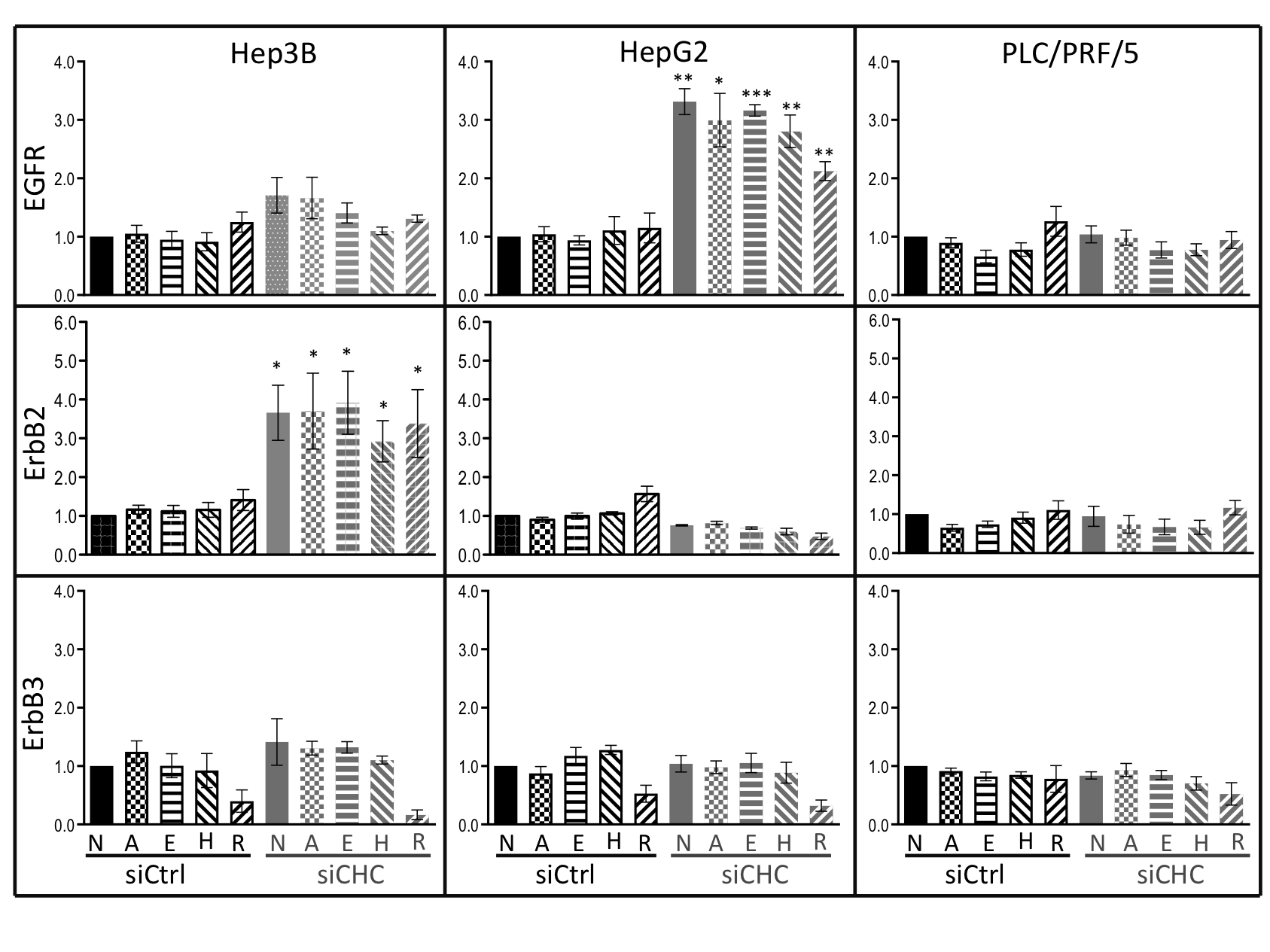

Supplement: Supplementary file 1 — Fig S1 [file JCMM-24-8091-s001.tif]

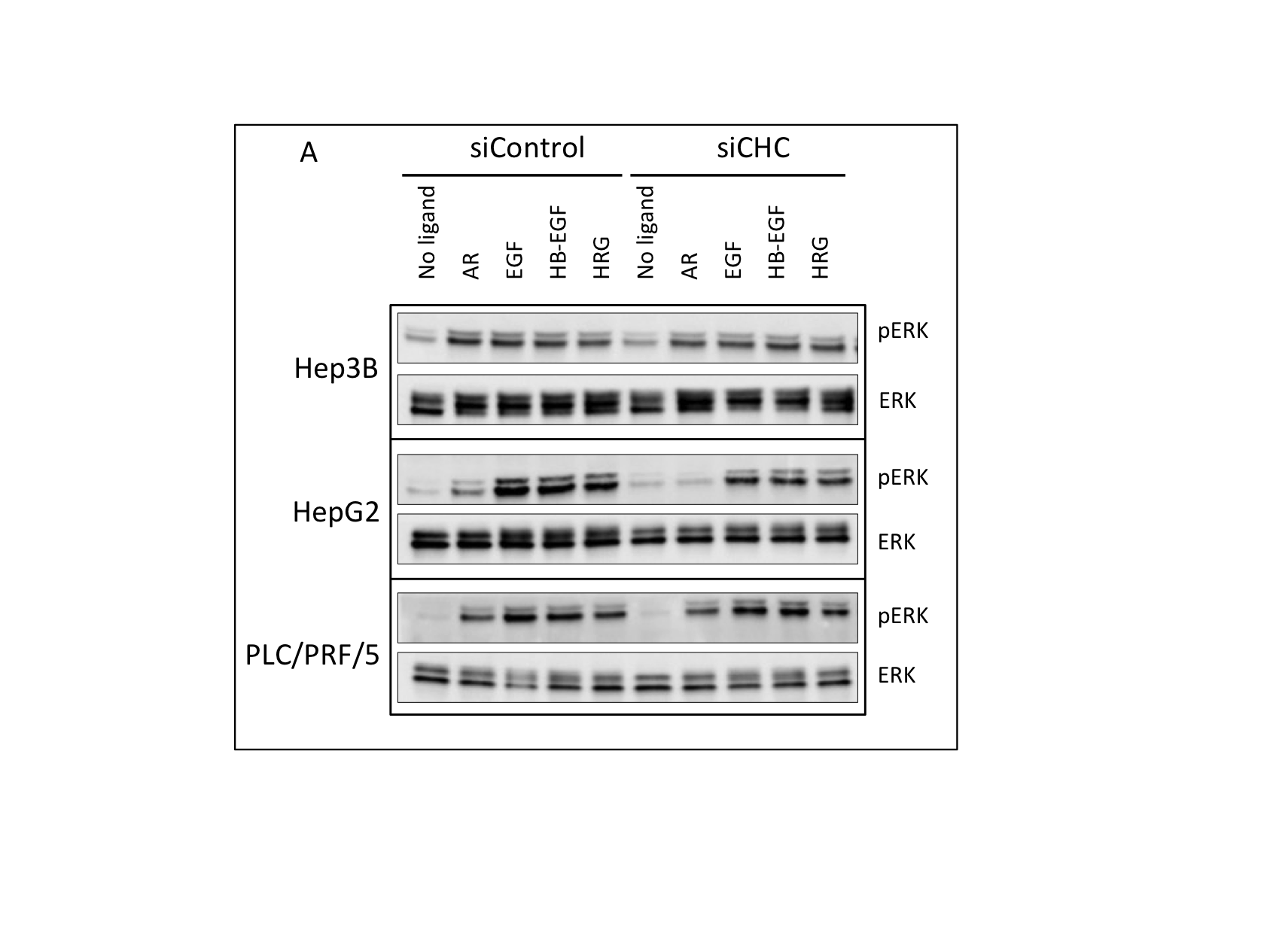

Supplement: Supplementary file 2 — Fig S2 [file JCMM-24-8091-s002.tif]
