# Supplementary material for: Regulation of the EGFR/ErbB signalling by clathrin in response to various ligands in hepatocellular carcinoma cell lines
Source: J Cell Mol Med. 2020 Jun 9;24(14):8091–102. doi: 10.1111/jcmm.15440 (PMC7348188; doi:10.1111/jcmm.15440)
Supplement: Supplementary file 3 — Table S1 [file JCMM-24-8091-s003.docx]

|  | **D5S818** | **D7S820** | **D13S317** | **D16S539** | **Amelogenin** | **CSF1PO** | **THO1** | **TPOX** | **vWA** |
| --- | --- | --- | --- | --- | --- | --- | --- | --- | --- |
|  |  |  |  |  |  |  |  |  |  |
| **Hep 3B** | 13 | 8, 10 | 12, 14 | 10 | X | 8 | 6, 7 | 9 | 17 |
| **HepG2** | 11, 13 | 10 | 9, 13 | 12, 13 | X, Y | 10, 11 | 9 | 8, 9 | 17 |
| **PLC/PRF/5** | 12 | 9, 11 | 11, 12 | 13 | X | 10 | 8 | 8 | 15, 16 |
|  |  |  |  |  |  |  |  |  |  |

**Supplementary Table 1. DNA short tandem repeat profiles of the cell lines used**

Profiling was performed using the eight short tandem repeat (STR) markers recommended by the American Type Cell Culture (ATCC), which enables a 1 in 108 discrimination rate for unrelated individuals. Alleles are referred to by their number of repeats defined using a panel of six cell lines used as reference. The profiles of all three cell lines were identical to those available on the ATCC website.
